# hCoCena: horizontal integration and analysis of transcriptomics datasets

**DOI:** 10.1093/bioinformatics/btac589

**Published:** 2022-08-26

**Authors:** Marie Oestreich, Lisa Holsten, Shobhit Agrawal, Kilian Dahm, Philipp Koch, Han Jin, Matthias Becker, Thomas Ulas

**Affiliations:** Modular High Performance Computing and Artificial Intelligence, Deutsches Zentrum für Neurodegenerative Erkrankungen (DZNE), 53127 Bonn, Germany; Systems Medicine, Deutsches Zentrum für Neurodegenerative Erkrankungen (DZNE) e.V., 53127 Bonn, Germany; Systems Medicine, Deutsches Zentrum für Neurodegenerative Erkrankungen (DZNE) e.V., 53127 Bonn, Germany; Deutsches Zentrum für Neurodegenerative Erkrankungen (DZNE) e.V., PRECISE Platform for Genomics and Epigenomics at DZNE and University of Bonn, 53127 Bonn, Germany; Systems Medicine, Deutsches Zentrum für Neurodegenerative Erkrankungen (DZNE) e.V., 53127 Bonn, Germany; Genomics and Immunoregulation, LIMES-Institute, University of Bonn, 53115 Bonn, Germany; Systems Medicine, Deutsches Zentrum für Neurodegenerative Erkrankungen (DZNE) e.V., 53127 Bonn, Germany; Deutsches Zentrum für Neurodegenerative Erkrankungen (DZNE) e.V., PRECISE Platform for Genomics and Epigenomics at DZNE and University of Bonn, 53127 Bonn, Germany; Systems Medicine, Deutsches Zentrum für Neurodegenerative Erkrankungen (DZNE) e.V., 53127 Bonn, Germany; Deutsches Zentrum für Neurodegenerative Erkrankungen (DZNE) e.V., PRECISE Platform for Genomics and Epigenomics at DZNE and University of Bonn, 53127 Bonn, Germany; Science for Life Laboratory (SciLifelab), KTH Royal Institute of Technology, Stockholm 17165, Sweden; Modular High Performance Computing and Artificial Intelligence, Deutsches Zentrum für Neurodegenerative Erkrankungen (DZNE), 53127 Bonn, Germany; Systems Medicine, Deutsches Zentrum für Neurodegenerative Erkrankungen (DZNE) e.V., 53127 Bonn, Germany; Systems Medicine, Deutsches Zentrum für Neurodegenerative Erkrankungen (DZNE) e.V., 53127 Bonn, Germany; Deutsches Zentrum für Neurodegenerative Erkrankungen (DZNE) e.V., PRECISE Platform for Genomics and Epigenomics at DZNE and University of Bonn, 53127 Bonn, Germany; Genomics and Immunoregulation, LIMES-Institute, University of Bonn, 53115 Bonn, Germany

## Abstract

**Motivation:**

Transcriptome-based gene co-expression analysis has become a standard procedure for structured and contextualized understanding and comparison of different conditions and phenotypes. Since large study designs with a broad variety of conditions are costly and laborious, extensive comparisons are hindered when utilizing only a single dataset. Thus, there is an increased need for tools that allow the integration of multiple transcriptomic datasets with subsequent joint analysis, which can provide a more systematic understanding of gene co-expression and co-functionality within and across conditions. To make such an integrative analysis accessible to a wide spectrum of users with differing levels of programming expertise it is essential to provide user-friendliness and customizability as well as thorough documentation.

**Results:**

This article introduces horizontal CoCena (hCoCena: horizontal construction of co-expression networks and analysis), an R-package for network-based co-expression analysis that allows the analysis of a single transcriptomic dataset as well as the joint analysis of multiple datasets. With hCoCena, we provide a freely available, user-friendly and adaptable tool for integrative multi-study or single-study transcriptomics analyses alongside extensive comparisons to other existing tools.

**Availability and implementation:**

The hCoCena R-package is provided together with R Markdowns that implement an exemplary analysis workflow including extensive documentation and detailed descriptions of data structures and objects. Such efforts not only make the tool easy to use but also enable the seamless integration of user-written scripts and functions into the workflow, creating a tool that provides a clear design while remaining flexible and highly customizable. The package and additional information including an extensive Wiki are freely available on GitHub: https://github.com/MarieOestreich/hCoCena. The version at the time of writing has been added to Zenodo under the following link: https://doi.org/10.5281/zenodo.6911782.

**Supplementary information:**

Supplementary data are available at *Bioinformatics* online.

## 1 Introduction

While the newest generation of sequencing technologies has led to a wealth of available transcriptomic datasets in the past years, the design of these datasets is mostly focused on answering distinct scientific questions, restricting the experimental setup to the respective questions. This is mostly due to high study costs and the lack of sample availability, especially in the field of human biology (Dumas-Mallet *et al.*, 2017), where sampling depends on patient availability and their willingness to engage in biomedical studies. Meanwhile, it is becoming clear that gaining a more holistic and systematic understanding of the gene expression landscape necessitates not only looking at single transcriptome studies and datasets but also combining information from multiple studies to increase information content while preventing the expenses of large studies on a single research team (Moretto *et al.*, 2019). This illustrates the need for tools that facilitate the integration of numerous transcriptome datasets, allowing researchers to combine multiple single studies into a comprehensive dataset for more holistic knowledge discovery. The process of combining several omics datasets that measure the same biomolecular entity—genes, in the case of transcriptomics—across different sample spaces has been referred to as horizontal data integration (Ulfenborg, 2019). A frequently discussed issue of dataset integration is the inconsistency of dimensionalities in both the number of samples and the number of genes measured. To facilitate the integration process, several methods have been proposed, one of which is referred to as *transformation-based integration* (Ritchie *et al.*, 2015). Here, all datasets are first separately transformed into a common structure which then provides the basis for the integration. This allows for the combination of datasets that vary in their dimension of the feature and sample space. The approach of transformation-based integration is the method underlying the presented work.

The data integration strategy used in hCoCena is based on weighted co-expression networks. Networks have been used extensively to model biological data (Jardim *et al.*, 2019; Langfelder and Horvath, 2008; Lemoine *et al.*, 2021; Marwah *et al.*, 2018; Pavel *et al.*, 2021; Proost and Mutwil, 2018; Russo *et al.*, 2018) and they often serve as the common structure used in transformation-based integrations. In the context of co-expression, the vertices represent genes, while the edges represent the co-expression of a pair thereof. In hCoCena, the edges are weighted, with the weights indicating correlation strength. Representing genes and their relationships in the form of networks enables the detection of underlying structures using community detection algorithms, without extensively depending on the original data type. This makes networks especially interesting in the context of multi-omics data integration.

Not only is there an increasing need for tools that enable multi-study transcriptomic data integration, but these tools also need to be provided in a format that facilitates application by researchers with entry-level programming skills and computer science knowledge while still remaining flexible and expandable for those with an extended programming background. A commonly seen issue with released tools and packages in the context of omics data analysis is their polarized implementation: thorough documentation, intended to increase user-friendliness, often comes at the cost of a linear analysis design that poorly adapts to different study layouts and research questions. On the other hand, packages that allow for great flexibility and combination with other methods are oftentimes scarcely documented, making them difficult to use, especially for users that are not in-depth familiar with the used programming language. This is particularly problematic in the case of omics data analyses since many researchers have a high-level knowledge of natural sciences, rather than computer science. hCoCena successfully provides a balance between these poles by choosing a modularly designed library paired with thorough documentation of functions, in- and outputs, and a pre-implemented ready-to-use analysis workflow that can be easily adapted and expanded with user-written functions and scripts to fit the analysis to the question and the data at hand.

## 2 Materials and methods

Horizontal-CoCena includes and expands our previously introduced tool CoCena^2^ (Aschenbrenner *et al.*, 2021) that allows for the analysis of a single transcriptomic dataset, using a co-expression network for the identification of gene clusters and their subsequent functional analysis. hCocena is a completely remastered, stand-alone version of the original CoCena. It provides improved user-friendliness, increased performance and higher computational efficiency, due to rigorous restructuring of the code-base and optimized data handling. It additionally introduces new features to the analysis and includes the option to jointly analyse more than one dataset (here interchangeably also referred to as a *layer*).

hCoCena’s ready-to-use workflow implementation is provided as an R markdown file utilizing the package functions with minimal code exposure and detailed descriptions of all in- and outputs as well as function parameters. The design reflects the modular character of the analytic process, making it easy to add new custom steps in-between and therefore allowing advanced usage and flexibility. To further increase user-friendliness, the corresponding GitHub repository offers a full exemplary analysis showcasing the functionalities of the tool on real-data examples and providing guidelines for parameter selection. The tool entails highly organized data structures to make working with multiple datasets as intuitive and structured as possible, avoiding littering of the analysis environment and subpar variable naming. Instead, the data has been clearly organized into an hCoCena object (*hcobject*), collecting data and variables associated with specific steps in descriptive slots and giving each analysis a unified structure. An overview of the object structure and its contents is provided in the repository’s Wiki.

The inputs required to run hCoCena are (i) a normalized and—optionally but recommended by Parsana *et al.* (2019)—batch-corrected count matrix for each dataset with genes as rows and samples as columns, (ii) an annotation file for each dataset with samples as rows and columns containing different categories of meta information and (iii) if the respective enrichment is desired—.gmt files for Hallmark, Gene Ontology (GO), Kyoto Encyclopedia of Genes and Genomes (KEGG) and Reactome enrichment (a ready-to-use set of files is provided for download in the repository). Overall, the tool is separated into a main markdown and satellite markdowns. The main markdown forms the backbone of the analysis and is roughly divided into three phases (see below) while the satellite markdowns contain a collection of optional additional analysis steps that the user can run if desired and which are easily extendable.

To address the persistent issue of reproducibility of scientific findings (Yu and Hu, 2019), the complete analysis can be exported to an HTML file including the results and the values of all set parameters. Thus, only access to the data must be provided to guarantee the reproduction of all results, increasing the transparency and credibility of published work. The figures produced during the workflow are of publication-ready quality to minimize the additional workload needed to close the gap between data analysis and publication. They are mostly implemented in *ggplot2* (Wickham, 2016), therefore allowing easy modification and customization.

### 2.1 Main phases of the hCoCena workflow

**Fig. 1.**
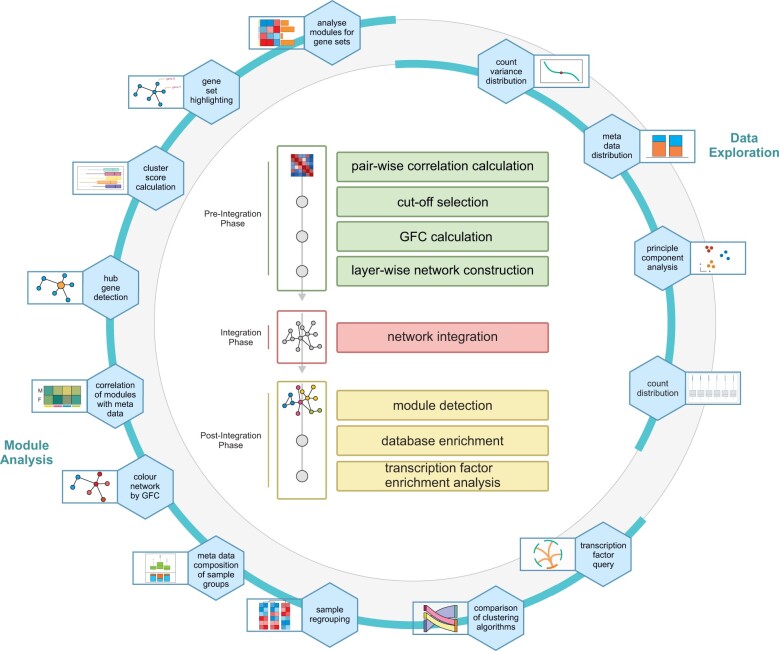
hCoCena overview. The main steps of the analysis backbone are shown in the centre. These functions are provided in the main markdown including descriptions and references to satellite functions. The ‘orbit’ around the central steps illustrates the available satellite functions. These are not part of the main script and can be added or left out of the analysis as desired. The user can also add custom functions to the pool of satellites. In general, the satellite functions form two groups: data exploration functions enable a first impression of the data at hand, while the other functions are part of the module analysis and can only be applied once the co-expression modules have been detected in the main analysis (Botía *et al.*, 2017)

A three-step concept has been chosen for the presented tool: the pre-integration phase, which contains pre-processing steps that are dataset-specific, followed by the network-based integration phase and the post-integration phase, which conducts different cross-layer analyses (Fig. 1).

#### Pre-integration phase

During the pre-integration phase, each layer separately undergoes five pre-processing steps: (i) extraction of the most variant genes, the number of which can be set by the user for each layer independently, optionally guided by a data-driven cut-off. This step may also be skipped; (ii) calculation of pairwise correlations for each gene pair per dataset, either using Pearson’s Correlation Coefficient, Spearman’s Rank Correlation Coefficient or previously calculated correlation values (e.g. using other tools); (iii) selection of a layer-specific correlation cut-off, which determines the minimum correlation required for a pair of genes to form an edge in the subsequently built network. The selection of this cut-off is aided by providing a series of statistical parameters that result from a variety of possible cut-off values, including criteria such as overall network size and the scale-free topology of the network (Carlson *et al.*, 2006; van Noort *et al.*, 2004); (iv) Group Fold-Change (GFC) computation for every gene per layer (details see below); and (v) co-expression network construction.

GFCs reflect the overall expression trend of a gene with respect to groups of samples that form disjoint subsets of the entirety of samples (e.g. a treatment, a disease). The calculation of the GFCs is different depending on the absence or presence of control samples in the datasets. In the case of no controls, let X be the set of unique sample labels in the dataset (e.g. *disease1* and *disease2*), with $x_{i}\in X$ denoting the *i*th label and $\mathrm{mean}\left(g_{x_{i}}\right)$ being the mean expression value of a gene g in the group of samples with label $x_{i}$, then the GFC of that gene for each condition is calculated as

$$\frac{\mathrm{mean}\left(g_{x_{i}}\right)}{\frac{1}{\left|X\right|}*\sum_{x_{i}\in X}\mathrm{mean}\left(g_{x_{i}}\right)}.$$

In the presence of control samples, let N be the total number of datasets, $x_{i,n}$ the *i*th non-control label in the condition space of dataset n ∈ 1, …, N, $x_{c,n}$ the group of control samples in dataset n and $\mathrm{mean}\left(g_{x_{i,n}}\right)$ be the mean expression value of a gene across a group of samples with label $x_{i}$ in dataset n. Then the GFCs of a gene for each non-control group i and each dataset n is calculated as

$$\frac{\mathrm{mean}\left(g_{x_{i,n}}\right)}{\mathrm{mean}\left(g_{x_{c,n}}\right)}.$$

When using multiple datasets that are not well-matched in sample sizes per condition or number of conditions, it is advised to use proper controls as a reference, such that hCoCena can correct for these differences.

#### Integration phase

During the integration phase, the co-expression networks constructed for the separate layers in the previous step are integrated. This transformation-based integration has been chosen to avoid the problems of differing data types and scales—as is the case when combining, e.g. RNA-Seq and Microarray data—as well as differing measurement inaccuracy and dataset-based batch effects. Differing levels of measurement inaccuracy would pose a particular problem if the data were integrated based on the original data structure. Datasets with larger measurement inaccuracy and, thus, increased variance due to technical rather than biological reasons, would predominate the signal from other datasets and heavily influence the grouping of genes into clusters, leading to a technical bias. This can be avoided by transforming the data from its original format to a network representation before integration (Ritchie *et al.*, 2015), which is done here. The nodes in the networks retrieved from each layer all represent the same type of biomolecule, that is, genes. Thus, an overlap of nodes is likely. In order to integrate the constructed networks, the user has the choice between two options: (i) integration by intersection, where the network of one of the datasets serves as a reference for the others, and the analysis is geared towards how the respective network changes in the other datasets; (ii) integration by union, where the resulting network is a union of the layer-specific networks and therefore provides a more holistic understanding of gene co-expression, utilizing all the information at hand and combining it into a single model. When choosing integration by union, many edges are expected to exist in multiple layer-specific networks. The user can then define if the integrated edge should have the minimum, mean or maximum weight of the edges in the layer-specific networks. Recommended is to choose the minimum to approximate the true co-expression using the lower boundary and—on a structural level—to facilitate the subsequent clustering of the network by reducing its density. The integrated network may be visualized using R-based layout functions or, alternatively, the network can be exported to Cytoscape (Shannon *et al.*, 2003).

#### Post-integration phase

The third phase is designed to functionally analyse the integrated network. The tool offers a variety of different clustering algorithms as provided by the R-packages *igraph* (Csardi and Nepusz, 2006) and *leidenAlg*, based on previously published resources (Traag *et al.*, 2019), to detect community structures (here interchangeably referred to as *modules* or *clusters*) in the network. The modules, consisting of genes with very similar expression patterns across samples, can be compared across conditions—i.e. sample labels—based on their mean GFC value and can then be further evaluated using enrichment analyses. Two types of enrichment analyses are offered in the main analysis workflow: (i) enrichments using public databases, such as GO (Ashburner *et al.*, 2000; The Gene Ontology Consortium, 2021), KEGG (Kanehisa and Goto, 2000), Hallmark molecular signatures database (Subramanian *et al.*, 2005) and the Reactome database (Gillespie *et al.*, 2022) and (ii) transcription factor target enrichment using the ChEA3 tool (Keenan *et al.*, 2019). Additional enrichment analyses can be run optionally in the satellite markdown, such as enrichment analyses based on user-defined gene sets or metadata enrichment based on provided sample annotation.

### 2.2 Additional analysis options

The co-expression analysis from the main markdown can be further expanded by a large variety of optional analysis steps presented in the satellite markdowns (Fig. 1). These allow for a further in-depth exploration of the data, letting the user go far beyond the conventional analysis of differentially expressed genes by using functionalities such as hub gene detection, principal component analysis, count distributions plots, meta-data correlation, gene set visualization, importing and exporting of generated network models, transcription factor querying and many others. These functions can be run independently and in no particular order. Dependencies on the progression through the main analysis are pointed out in the descriptions and the main markdown references the satellite functionalities at the most suitable steps throughout the workflow. The satellite functions have been detached from the analysis backbone to allow maximal flexibility and to easily add custom functions. All available satellite functions can be found in the repository’s Wiki pages.

## 3 Results

### 3.1 Integration strategy showcase

To showcase some functionalities of hCoCena and most importantly the robustness of the integration strategy, we selected two public datasets that describe a similar experimental setup but were sequenced using different technologies, namely Microarray and RNA-Seq. Details on data pre-processing and dataset availability can be found in the Supplementary Material. The integration of Microarray data with RNA-Seq data is challenging, despite the fact that tremendous efforts have been made to find ways of combining their data (van der Kloet *et al.*, 2020). Here, we demonstrate that the network-based data integration successfully overcomes these issues. The datasets used here comprise human samples and investigate a macrophage activation assay, describing stimulation with IFN-γ and IL-4 alongside no stimulation (*baseline*) (Beyer *et al.*, 2012). Macrophage activation covers a wide spectrum of activation states (Xue *et al.*, 2014), but broadly speaking, IFN-γ is known to induce a pro-inflammatory phenotype, while IL-4 induces an alternative, anti-inflammatory phenotype. We will not go into the biological details of the activation process, but rather use this data to showcase the reproducibility of known results while integrating data from vastly different technologies.

**Fig. 2.**
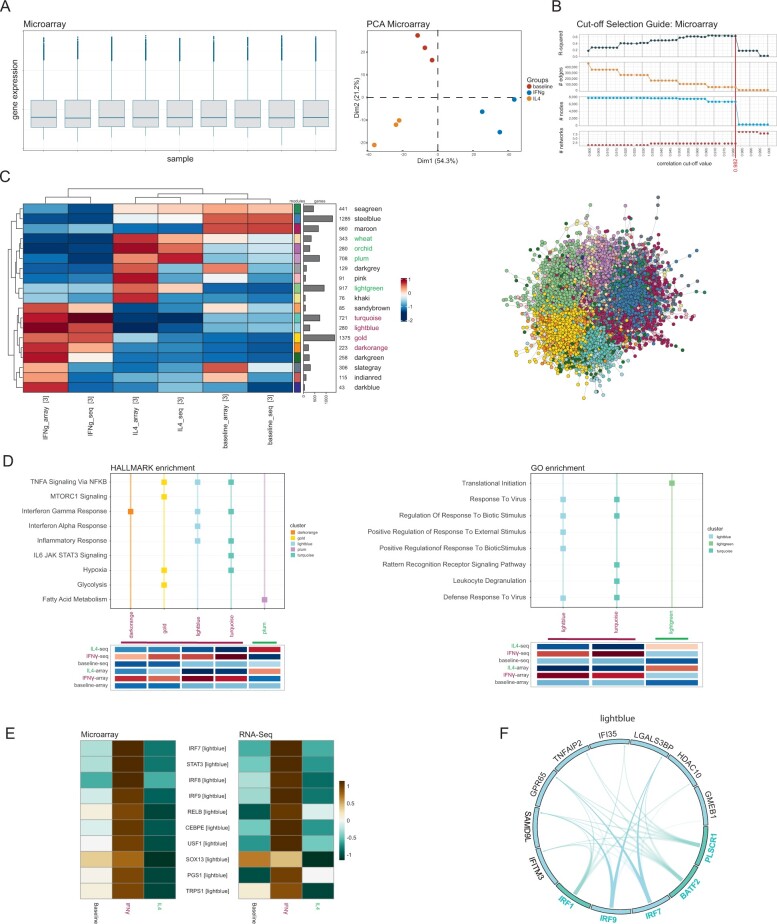
hCoCena results of the two integrated macrophage activation assays. (**A**) Initial data exploration, here exemplified on the Microarray data. Shown are the gene expression values per sample as boxplot (left) and a PCA of the samples coloured by treatment (right). (**B**) The cut-off selection interface provided to the user. It visualizes four different network criteria (top to bottom: *R*^2^, no. edges, no. nodes, no. networks) for a series of different correlation cut-off values. (**C**) The module heat-map (left) the shows the co-expression modules detected in the integrated network (right) as rows and the defined sample groups as columns. The cell colouring indicates the GFC value of the respective sample group across the genes of the corresponding module. Numbers and bar-plots on the right side indicate the sizes of the modules. (**D**) Exemplary Hallmark Molecular Signatures and Gene Ontology enrichments of selected modules. Shown are the top 5 most enriched terms with adjusted *P*-values ≤0.1. Module names are highlighted with respect to stimulus-specificity: IFN-γ in purple and Interleukin-4 in green. (**E**) Scaled mean expressions of hub genes detected for the *lightblue* module confirm IFN-γ-specific expression patterns. (**F**) Top5 transcription factors (TFs) with the most enriched targets on cluster *lightblue*. TFs are highlighted in turquoise font, their targets are in black. Cell colours indicate the module the genes belong to. Arcs indicate the TFs known targets, saturated arc colours represent that a corresponding edge is present in the integrated co-expression network

To get an initial impression of the data and to detect possible outliers, the expression-value distributions for all samples and a Principle Component Analysis were plotted for each dataset (Fig. 2A, Supplementary Fig. S1A). Both plots indicated no outliers or other data irregularities in either of the datasets. The Microarray data were filtered for the top 7700 and the RNA-Seq data for the top 7664 most variant genes, i.e. genes with the largest variance in expression value across samples, as suggested by the satellite function *suggest_topvar()* that finds a data-driven filtering threshold based on inflection points in the ranked log-variance curve. The motivation behind this is to remove genes with very little variance in their expression values across conditions, which implies low impact of the conditions on the genes’ expression levels, deeming them irrelevant for the phenotype. Spearman’s Rank Correlation was used to calculate the pair-wise co-expression values and the cut-off statistics were calculated for 50 cut-off values in the range 0.9 to 1.0 and visualized in the cut-off selection guides (Fig. 2B, Supplementary Fig. S1B). Both guides presented the highest network quality at a correlation cut-off of 0.982. The quality was evaluated by maximizing the scale-free topology (measured using the *R*^2^-value of the logged node-degree distribution) while keeping as many genes as possible and reducing the number of edges to avoid the ‘hairball’ effect: a too densely connected network that loses its structure and does not allow community detection. Based on this correlation cut-off, a co-expression network was constructed for each dataset. The networks were then integrated using the integration-by-union principle, hence also genes and edges that are only present in one but not the other dataset will be present in the integrated network. This gives not only an impression of the co-expressions that are shared among the two datasets but also of those that are unique to one or the other, Presenting a more wholistic picture. Multi-edges, i.e. edges that are present in both datasets but with different correlation weights, were simplified by using the lowest correlation value found in either of the datasets for the corresponding gene pair. This prevents over-estimating the importance of their co-expression and furthermore makes the network less dense, facilitating the identification of clusters. The integrated network consisted of 8336 nodes (i.e. genes) and 90 202 edges (i.e. correlations that exceeded the cut-off value). The network was clustered using the Leiden algorithm, yielding 19 modules. Eighteen genes were dropped because they were assigned to clusters smaller than the set minimum size of 25 genes. The integrated network coloured by module and the module heatmap showing the modules’ mean GFCs across conditions are shown in Figure 2C. The network layout was generated using the *Prefuse Force Directed Layout* of Cytoscape (version 3.8.2) (Shannon *et al.*, 2003) GFCs show clear stimulus-specific trends independent of the sequencing platform emphasizing their utility for the integration task (Supplementary Fig. S1C). The clusters *wheat*, *orchid*, *plum* and *lightgreen* show IL-4-specific up-regulation, while *turquoise*, *lightblue*, *gold* and *darkorange* are up-regulated only in IFN-γ stimulated samples. These clusters were used for GO and Hallmark enrichment analyses. The results were filtered for the 5 most significant terms with adjusted *P*-values ≤0.1 (Fig. 2D) per cluster. If clusters are missing in the plot, then no significantly enriched terms were found. Clusters that are up-regulated in IFN-γ stimulated samples show Hallmark enrichment of interferon response terms, hypoxia, glycolysis, and general inflammatory response, while these gene clusters are down-regulated in IL-4 stimulated samples, confirming the pro- and anti-inflammatory roles of the two stimuli. A similar picture is painted in the GO-enrichment where the IFN-γ up- and IL-4 down-regulated gene clusters bring up enrichment terms associated with immune response. We further checked the cluster assignment of genes identified in the reference analysis (Xue *et al.*, 2014) as IFN-γ-specific transcription factors (Supplementary Fig. S1D). The majority of them were assigned to the clusters *lightblue*, *turquoise* and *gold*, showing IFN-γ-specific activation. To showcase the hub-gene detection, we selected the *lightblue* cluster, since most of the genes listed by Xue *et al.* (2014) appeared in this cluster. hCoCena detected *IRF7* and *IRF9* to be among the strongest hubs (Fig. 2E), both of which were noted to be IFN-γ-specific transcription factors in the reference analysis (Xue *et al.*, 2014).

The transcription factor enrichment analysis performed with hCoCena confirms their role in IFN-γ stimulation further (Fig. 2F).

These results show that biological findings that have previously been made on Microarray data only, can be reconstructed while integrating data from RNA-Seq and Microarray, two vastly different data collection techniques, despite the low sample size (*n* = 9) in each dataset. This showcases the strength of the network-based integration approach and its ability to filter out biological signals and overcome dataset-specific differences.

To additionally demonstrate the advantage offered by data integration as compared to single dataset analysis, we integrated the data from a COVID-19 cohort with that of a sepsis cohort. Details regarding the datasets as well as their preprocessing are available in the Supplementary Material. In the early stages of the COVID-19 pandemic, phenotypic parallels became apparent between severe COVID-19 cases and sepsis (Li *et al.*, 2020). Data integration of gene-expression data allows more detailed insight into this initial observation, as we can show here. The parameters set in the analysis can be found in the Supplementary Material. The patients from the COVID-19 cohort were grouped based on disease severity: *COVID 1* are the most severe cases, with severity decreasing until *COVID 5*, which are considered as mild cases. *COVID 6* is the healthy control group. Supplementary Figure S2A shows the module heat-map created by hCoCena for the integrated network. The dendrogram on the top clusters severe COVID-19 cases (Group 1) together with deathly sepsis cases while the mild cases (Group 5) cluster most closely with samples from uncomplicate sepsis cases. The second most severe COVID-19 cases (Group 2) are most closely linked to severe sepsis and septic shock and the intermediate COVID-19 cases cluster together. The healthy controls (Group 6) are distinctly different from all disease cases. However, despite the proximity of the rather severe COVID-19 cases with sepsis, there are nonetheless distinct differences on the transcriptional level, as has been shown previously (Aschenbrenner *et al.*, 2021). Cluster *plum* is up-regulated in both, severe COVID-19 (Groups 1 and 2) and sepsis with a deadly outcome. The Hallmark enrichment (Supplementary Fig. S2B) shows inflammatory response terms for this cluster. However, cluster *orchid*, which is also associated with inflammation, comprises genes specifically up-regulated in the group ‘sepsis death’. Thus, the integration of the two datasets highlighted the phenotypic commonalities of the two diseases but was also able to identify groups of genes that differed between severe COVID-19 cases and severe sepsis cases.

### 3.2 Comparison to other tools

**Fig. 3.**
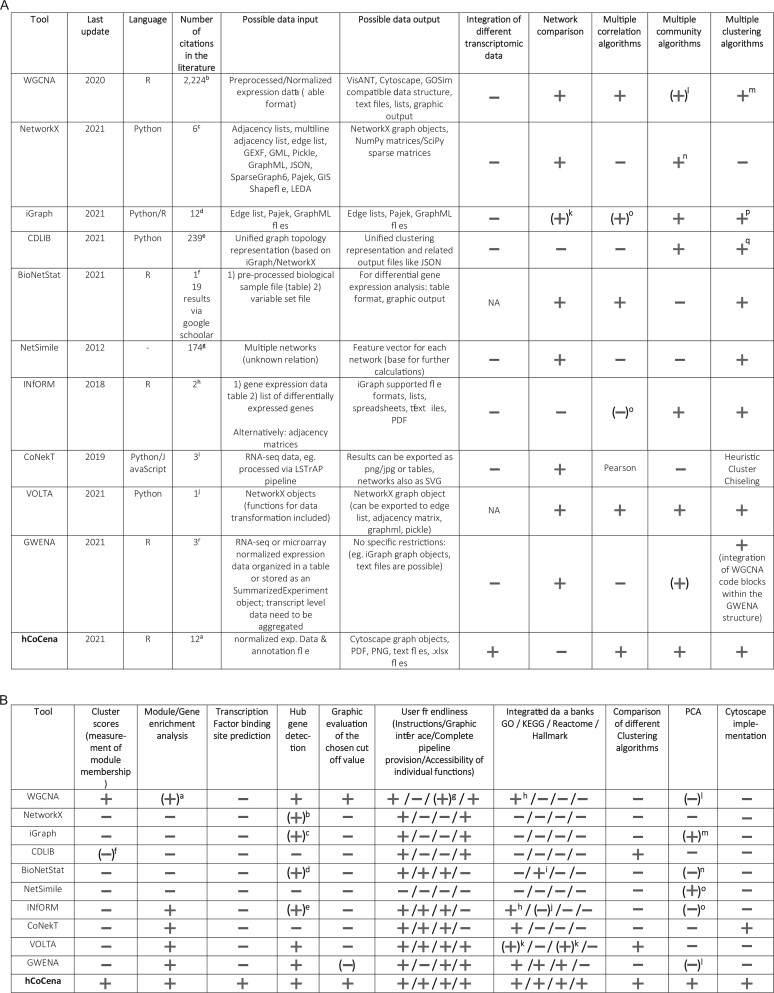
(**A**) Comparison of different network packages concerning general characteristics and included algorithms. All Pubmed and Google Scholar searches were performed at the October 15, 2021 with exception of the GWENA search (December 17, 2021); Multiple Correlation Algorithms refer to the options provided to analyse the correlation of genes. Multiple Community Algorithms refer to the algorithms provided to detect community structures (‘clusters’/‘modules’) in the network. Multiple Clustering Algorithms refer to the options provided to cluster data points, e.g. samples or sample groups. ^a^Google Scholar search ‘cocena2’; ^b^Pubmed search ‘WGCNA’; ^c^Pubmed search ‘networkx’ (eight results but two are not referring to NetworkX); ^d^Pubmed search ‘igraph’; ^e^Google Scholar search ‘CDlib (Community discovery library)’ since 2020, not all results refer to the CDLIB tool; ^f^Pubmed search ‘BioNetStat’; ^g^Google Scholar search ‘NetSimile’; ^h^Google Scholar search ‘INfORM: Inference of NetwOrk Response Modules’ (both entries are referring to the same paper); ^i^Pubmed search ‘CoNekT’; ^j^Google Scholar search ‘VOLTA (advanced molecular network analysis)’; ^k^Hamming distance can be calculated taking specific conditions into account; ^l^in general the module detection is enabled via unsupervised clustering, by default hierarchical clustering dendrogram and branch cutting is used; ^m^WGCNA pipeline can be supplemented by other algorithms like k-means clustering (Botía *et al.*, 2017); ^n^example for the implemented algorithms: Louvain & Tree partitioning; ^o^they can make use of the base R function cor(); ^p^clustering algorithms like cluster_louvain, cluster_fast_greedy, cluster_edge_betweenness are included and are used for community detection; ^q^different clustering approaches (like NodeClustering, FuzzyNodeClustering) are included for the standardized representation of community structures; ^r^Google Scholar search ‘GWENA Lemoine’ (11 results in total, 3 could be associated with the discussed R package). (**B**) Comparison of different network packages concerning features, user-friendliness and connected data banks. ^a^A correlation between modules and provided numerical traits can be performed, further enrichment analysis can be performed via recommended online tools like David; ^b^degree centrality of the graph components can be measured; ^c^connecting strength of graph components can be calculated; ^d^centrality analysis can be used to highlight key genes; ^e^node importance can be calculated and used for evaluation; ^f^FuzzyNodeClustering function also includes the generation of a ‘node-community allocation probability matrix to keep track of the probabilistic component of the final non-overlapping partition’ (Rossetti *et al.*, 2019); ^g^provided tutorials are guiding through complete analysis pipelines, the code chunks have to be adjusted manually; ^h^Gene ontology enrichment analysis can directly be performed within R using GO.db; ^i^the required data are provided using pathview R package; ^j^INfORM can make use of GSEABase package; ^k^GO enrichment API can be used ‘to perform enrichment against Reactome Pathways as well as GO or the Panther Protein class’ (https://github.com/fhaive/VOLTA/blob/master/jupyternotebooks/Example_of_Enrichment.ipynb); ^l^PCA is not performed like in hCoCena but the module eigengene can be calculated which is the first principle component of the expression matrix; ^m^Spectral Coarse Graining can be performed, ‘(PCA) can be viewed as a particular SCG, called exact SCG, where the matrix to be coarse-grained is the covariance matrix of some dataset’ (igraph R manual pages); ^n^PCA like hCoCena cannot be performed but eigenvectors can be calculated; ^o^graph/feature matrix can be projected into the principle component space via single value decomposition

A plethora of different network analysis tools exist that can be used to investigate gene co-expression networks and more are released frequently (Csardi and Nepusz, 2006; Falousos, 2012; Hagberg, 2008; Jardim *et al.*, 2019; Langfelder and Horvath, 2008; Lemoine *et al.*, 2021; Marwah *et al.*, 2018; Pavel *et al.*, 2021; Proost and Mutwil, 2018; Rossetti *et al.*, 2019). To facilitate the comparison between existing tools and to emphasize the large variety of analysis options provided by hCoCena, Figure 3 provides an overview in tabular format comparing the main features of these tools and the programming languages that they are implemented in, the input and output format of data, if data integration is available and if so, which strategy has been used, and many other aspects. It becomes evident that although so many tools are available, they are complementary rather than exhaustive in their offered functionalities. hCoCena, however, unites an unprecedented number of analysis options into one single tool.

## 4 Conclusion

The wealth of transcriptomic datasets that has been accumulated over the past years and continues to increase has demanded the development of tools that allow the integrative analysis of this data. With hCoCena, we offer a solution to this problem, by combining different transcriptomic datasets using a network-based integration approach. The tool further provides the user with a wide variety of downstream analysis applications and makes it easy to add additional functionalities specifically designed for the data at hand.

hCoCena is the first steps to what is intended to become a tool suite for omics data analysis offering options for single- and multi-set analyses—as now provided by hCoCena—as well as planned extensions for single-cell data, multi-omics-data integration, and clinical study-oriented analysis. The suite is intended to assist researchers in easily tailoring an analysis workflow to their experimental setup, especially in the prospect of increased utilization of multi-omics technologies and the combined evaluation of the retrieved data.

## Supplementary Material

btac589_Supplementary_Data
